# Municipal implementation of community-based preventive programs during the COVID-19 pandemic and long-term care certification trends in Japan: a nationwide ecological analysis

**DOI:** 10.1186/s12913-026-14909-0

**Published:** 2026-06-06

**Authors:** Yujiro Kuroda, Masatsugu Orui, Yumi Shindo, Yohei Koyama, Kohta Suzuki

**Affiliations:** 1 Department of Health and Psychosocial Medicine, School of Medicine, Aichi Medical University, 1-1 Yazakokarimata, Nagakute, 480-1195 Aichi Japan; 2 International Research Institute of Disaster Science, Tohoku University, Sendai, Miyagi Japan; 3 Tokyo Metropolitan Institute for Geriatrics and Gerontology, Itabashi-ku, Tokyo, Japan; 4 National Center for Geriatrics and Gerontology, Obu, Aichi Japan; 5 Department of Public Health, Fukushima Medical University, Fukushima- shi, Fukushima, Japan

**Keywords:** Long-term care, Preventive services, Program implementation, COVID-19 pandemic, Ecological analysis, Aging, Municipalities, Implementation

## Abstract

**Background:**

The coronavirus disease (COVID-19) pandemic disrupted community-based preventive services for older adults. However, population-level evidence linking municipal implementation of such services during the pandemic to subsequent care-need outcomes remains limited. Japan’s long-term care insurance (LTCI) system, in which municipalities administer preventive programs and care-need certification is conducted through a standardized process, provides an appropriate setting for examining this association. This study examined whether municipal implementation status of preventive programs planned for FY2020 was associated with subsequent trends in LTCI certification rates.

**Methods:**

We conducted a nationwide ecological analysis linking a municipal survey conducted in 2021 to annual LTCI certification rates for FY2018–2023. The exposure was full versus incomplete implementation of programs planned for FY2020 across five prevention domains: physical activity, social participation, cognitive training, nutrition, and health check-ups. Collapsed difference-in-differences models compared changes in certification rates from the pre-pandemic period (FY2018–2019) to the pandemic (FY2020–2021) and post-pandemic (FY2022–2023) periods. Event-study models estimated year-specific differences in certification-rate changes. Models were adjusted for baseline LTCI certification rate, aging rate, log-transformed population size, and fiscal capacity index. Sensitivity analyses assessed additional contextual adjustment, exclusion of municipalities without planned programs, alternative LTCI certification outcomes, and potential nonresponse bias.

**Results:**

Of 1,741 municipalities invited, 1,114 were included in the primary analysis. Full implementation was reported by 174 municipalities (15.6%) for physical activity and 158 (14.2%) for cognitive training. Full implementation of physical activity programs was associated with smaller increases in LTCI certification rates during the pandemic period compared with incomplete implementation (-0.12% points [pp], 95% confidence intervals [CI]: -0.23 to -0.01). A comparable association was observed for cognitive training (-0.14 pp, 95%CI: -0.25 to -0.03). Sensitivity analyses generally showed similar directional patterns, although estimates were modest and less precise when additional contextual adjustment was applied, municipalities without planned programs were excluded, or alternative certification thresholds were used.

**Conclusions:**

In this nationwide ecological analysis, full implementation of physical activity and cognitive training programs planned for FY2020 was associated with smaller subsequent increases in municipal LTCI certification rates. These findings suggest that municipal implementation status during the COVID-19 pandemic may be related to population-level care-need trends. However, causal interpretation is limited by the ecological design, single-time assessment of implementation status, potential exposure misclassification, and possible pandemic-related changes in LTCI certification procedures.

**Supplementary Information:**

The online version contains supplementary material available at 10.1186/s12913-026-14909-0.

## Background

The coronavirus disease (COVID-19) pandemic disrupted preventive health services across the globe, particularly those delivered through community-based, face-to-face formats for older adults [1]. The World Health Organization documented widespread service disruptions in over 90% of surveyed countries, with preventive and promotive services among those most frequently affected.

Although the indirect health consequences of service disruption, including delayed diagnoses, deconditioning, and accelerated functional decline, have been widely discussed [2, 3], population-level evidence linking municipal implementation of community-based preventive services during the pandemic to subsequent disability-related administrative outcomes remains limited. Most studies have relied on individual-level surveys of care avoidance or self-reported health decline [4], and few have examined whether variation in municipal implementation status was associated with subsequent trends in formally assessed care needs.

Japan is the world’s most aged society, providing an informative setting for addressing this question. Since 2000, the country has operated a universal long-term care insurance (LTCI) system in which municipalities serve as both insurers and local administrative bodies responsible for planning and implementing long-term care prevention programs [5, 6]. These programs span multiple domains, including physical activity, cognitive training, social participation, nutrition, and health check-ups, and are delivered under a shared national policy framework [7, 8]. Eligibility for formal LTCI services is determined through a nationally standardized certification process that integrates assessor-administered evaluations of activities of daily living, cognition, and behavior with a primary physician’s opinion, reviewed by a municipal committee [5]. The resulting certification rate serves as an administrative disability indicator at the population level and has been used extensively in epidemiologic research [6, 9]. Functionally analogous systems exist in other countries with statutory long-term care insurance, including Germany’s Pflegegrad assessment [10] and the Republic of Korea’s long-term care grading system [11], in which standardized needs assessments determine eligibility and benefit levels. The Japanese system is distinctive because both the preventive service delivery and disability assessment are administered by the same municipal authority, creating a nationally uniform structure within which local variation in implementation can be systematically examined.

Prior studies have examined related but distinct forms of preventive activity, including LTCI prevention services, social participation, and community gathering places such as kayoi-no-ba. Participation in LTCI prevention services may be effective among specific groups of older adults, although effects may not be uniform across all individuals [12]. Social participation, particularly involvement in multiple types of community organizations, has been associated with a lower risk of incident functional disability among community-dwelling older Japanese adults [13]. Community-based salon-type gathering programs (kayoi-no-ba), introduced through municipal social-participation interventions, have also been associated with reduced subsequent functional disability [14]. However, these observational studies may have been affected by selection and endogeneity, because individuals who participate in such activities may differ systematically from non-participants in health status, motivation, social resources, or access to services. Cognitive training interventions have demonstrated improvements in memory and executive function, although the magnitude and durability of effects vary across individuals [15, 16]. Nutritional support and routine health check-ups may play complementary roles by addressing frailty and facilitating early detection of health risks [17]. However, most evidence is derived from cohort studies or trials conducted under stable conditions, and the extent to which these benefits are preserved or lost, when service delivery is disrupted by external shocks has not been evaluated at the population level.

The COVID-19 pandemic substantially disrupted the delivery of community-based prevention programs in Japan and elsewhere. Infection control measures, including guidance to avoid the “three Cs” (closed spaces, crowded places, close-contact settings) [18, 19], led many municipalities to suspend or scale back group-based activities, whereas transitions to remote or modified formats were uneven and often infeasible for older populations with limited digital literacy [20, 21]. These disruptions created inter-municipal variation in the implementation status of preventive programs planned for FY2020 across prevention domains. From a health services research perspective, this variation provides an opportunity to examine whether municipalities reporting full implementation of planned programs experienced different subsequent LTCI certification-rate trends compared with municipalities reporting incomplete implementation [22]. However, because implementation status was measured at a single survey point and was not randomly assigned, the analysis should be interpreted as an ecological comparison rather than a definitive causal evaluation. Although national statistics indicated changes in LTCI certification during this period [23], aggregated data obscure variation in municipal implementation status across municipalities and intervention domains. Thus, whether full implementation of preventive programs planned for FY2020 was associated with subsequent trends in LTCI certification rates remains unclear.

To address this gap, we linked data from a nationwide municipal survey conducted in late 2021 with administrative LTCI certification data for fiscal years (FY) 2018–2023. By classifying implementation status in each prevention domain as “full” or “incomplete” and applying collapsed difference-in-differences (DiD) and event-study analyses, we aimed to examine whether full implementation of community-based preventive programs planned for FY2020 was associated with subsequent changes in LTCI certification rates during and after the COVID-19 pandemic. Our findings contribute to the evidence base on the population-level consequences of preventive service disruption by using a setting in which standardized outcome assessment, nationally uniform program design, and decentralized implementation allow municipal-level ecological analysis.

## Methods

### Study design and participants

This nationwide, municipality-level longitudinal study was conducted in Japan. Municipalities are the primary administrative units responsible for planning and implementing LTC prevention programs under the national LTCI system [6]. To capture program implementation during the COVID-19 pandemic, a cross-sectional survey was distributed to 1,741 municipalities between November and December 2021 to assess program status across five prevention domains using standardized response options. Responses were obtained from 1,120 municipalities (64.3%). After excluding 6 municipalities with missing outcome or explanatory variables, 1,114 were included in the analytical dataset. Survey data were linked to annual LTC certification statistics for FY 2018‒2023, enabling longitudinal analyses of certification trends in relation to FY2020 program implementation status. This study was conducted and reported in accordance with the Strengthening the Reporting of Observational Studies in Epidemiology (STROBE) guidelines for observational studies [24].

### Data sources

Annual LTC certification statistics were obtained from the *Long-Term Care Insurance Status Report* published by the Ministry of Health, Labor and Welfare (MHLW). Baseline municipal characteristics, including population size, aging rate, and fiscal capacity index, were retrieved from the e-Stat government database. Prefecture-level COVID-19 burden, defined as the average number of confirmed cases per 100,000 population, was derived from MHLW reports. Service infrastructure was assessed based on the number of general community support centers in each municipality [25].

### Variables

#### Exposure

The survey was conducted between November and December 2021, and municipalities were asked to report the implementation status of preventive programs planned at the beginning of FY2020. Respondents indicated whether programs were delivered as planned, partly delivered, or canceled during FY2020. Implementation status was assessed across five domains of community-based prevention: physical activity, social participation, cognitive training, nutrition, and health check-ups. For each domain, respondents selected one of four categories: “planned and fully implemented,” “planned and partly implemented,” “planned but canceled,” or “not planned.” Programs modified in response to the pandemic—such as reduced frequency, shortened duration, limited attendance, or online delivery—were classified as “fully implemented” if delivered as intended. For analysis, implementation was dichotomized as “full” or “incomplete” (partly implemented, canceled, or not planned).

Illustrative activities were based on examples provided in the MHLW *Kaigo Yobo Manual* (Manual for Long-term Care Prevention) [26], which has guided nationwide program development. These activities included exercise classes and strength training (physical activity), community salons and volunteer groups (social participation), memory quizzes and brain training (cognitive training), shared cooking or meal provision (nutrition), and routine health or cognitive check-ups (health check-ups).

#### Outcome

The primary outcome was the annual LTC certification rate, defined as the proportion of residents aged ≥ 65 years who were certified at Support Level 1 or higher. Under the Japanese LTCI system, certification is determined through a standardized process that includes assessment by a trained assessor, the opinion of a primary physician, and review by a municipal certification committee, providing an objective and reproducible measure of functional decline [6]. Certification status determines eligibility for formal LTC services [27] and has been widely used as an indicator of disability and frailty in epidemiologic studies [6, 9]. This study focused on certification at any level (Support Level 1 or higher), rather than stratifying by care level, to capture the onset of formal care needs at the population level. For each municipality, certification rates were calculated annually from FY2018 through FY2023.

#### Covariates

Adjustment variables included baseline LTC certification rate (mean of FY2018–2019), aging rate (percentage of residents aged ≥ 65 years), log-transformed population size, and fiscal capacity index. The fiscal capacity index, calculated as the 3-year average of standard fiscal revenue divided by standard fiscal expenditure needs, reflects municipal financial strength.

### Statistical analysis

We used two complementary analytic approaches: collapsed DiD models and event-study models. Collapsed DiD models were used to compare changes in LTCI certification rates between municipalities with full versus incomplete program implementation across three periods: pre-pandemic (FY2018–2019), pandemic (FY2020–2021), and post-pandemic (FY2022–2023). Implementation status was measured using the 2021 municipal survey and treated as the FY2020 implementation status of programs planned at the beginning of FY2020. The collapsed DiD models were used to estimate differences in changes in certification rates associated with full versus incomplete implementation, rather than annually varying program availability. Event-study models were used to regress year-specific deviations from the baseline certification rate, defined as the mean of FY2018–2019, on full implementation status, to allow estimating the year-specific differences and visual inspection of annual patterns before, during, and after the pandemic [28, 29]. Because implementation status was not randomly assigned but measured at a single survey point, these analyses should be interpreted as adjusted ecological comparisons rather than as definitive causal estimates.

All primary models were adjusted for baseline LTCI certification rate, aging rate, log-transformed population size, and fiscal capacity index. We conducted sensitivity analyses to assess robustness to additional contextual adjustment, exposure classification, outcome definition, and potential nonresponse bias. First, we repeated the collapsed DiD models with additional adjustment for municipal service infrastructure, measured as the number of community general support centers per 100,000 population, and prefecture-level COVID-19 burden, measured as the average daily number of newly confirmed COVID-19 cases per 100,000 population. Because data on infection were not consistently available at the municipal level, prefecture-level measures were used as proxies. Second, to examine whether the findings were influenced by municipalities that had not planned the relevant program at the beginning of FY2020, we repeated the collapsed DiD analyses after excluding municipalities that reported “not planned” for each domain. Third, to assess robustness to outcome definition, we repeated the analyses using two alternative LTCI certification outcomes: certification at Care Level 1 or higher and certification at Care Level 2 or higher. These models were adjusted for the baseline value of the corresponding outcome. Finally, to assess potential nonresponse bias, we compared responding and non-responding municipalities using publicly available demographic, fiscal, and baseline LTCI certification indicators.

Estimates are presented as percentage point (pp) differences with 95% confidence intervals (CIs). Robust standard errors were used for collapsed DiD and sensitivity analyses. For event-study models using repeated municipality-year observations, standard errors were clustered at the municipal level. Statistical analyses were performed using SPSS version 30 (IBM Corp, Armonk, NY, USA) and Python version 3.13 (Python Software Foundation, Wilmington, DE, USA).

### Ethical considerations

This study used municipality-level aggregated data without individual identifiers and therefore fell outside the scope of formal review by an institutional ethics committee under the regulations of the Ethics Committee of the National Center for Geriatrics and Gerontology. The municipal survey was conducted with permission from the Director of the National Center for Geriatrics and Gerontology. Designated municipal officers were provided with written information describing the purpose of the study, voluntariness of participation, data handling procedures, privacy safeguards, potential benefits and risks, and dissemination plans. Written informed consent was obtained by requiring participants to indicate their agreement on the questionnaire before participation. The study was conducted in accordance with the principles of the Declaration of Helsinki.

## Results

Of the 1,741 municipalities invited to participate in the late-2021 nationwide survey, 1,120 (64.3%) responded. After excluding 6 municipalities with missing outcomes or explanatory variables, 1,114 were included in the analytic cohort. Linkage to LTC certification statistics was complete for FY2018–2023, providing uninterrupted follow-up through March 2024 with no additional attrition.

Table 1 summarizes the baseline characteristics of the included municipalities, stratified by implementation of physical activity programs, the most widely implemented domain, and primary focus of policy and analysis. Most municipalities were small, with populations of < 100,000. The mean aging rate was 34.0%, and the baseline LTC certification rate averaged 17.6%, with similar values across the implementation groups. The distribution of service infrastructure, including general community support centers, varied modestly across strata.

**Table 1:** Baseline characteristics of municipalities participating in the 2021 nationwide survey (during the COVID-19 pandemic), stratified by implementation status of physical activity programs

Characteristic	All	Not planned/no implementation	Partial implementation	Full implementation
Number of municipalities, *n* (%)	1,114 (100.0%)	49 (4.4%)	891 (80.0%)	174 (15.6%)
Population size				
≤100,000 (small)	895	42 (4.7%)	711 (79.4%)	142 (15.9%)
100,000–199,999 (medium)	111	4 (3.6%)	93 (83.8%)	14 (12.6%)
≥200,000 (core city)	108	3 (2.8%)	87 (80.6%)	18 (16.7%)
Aging rate (% ≥65 years, mean SD)	34.0 (7.9)	34.8 (9.6)	34.0 (7.8)	34.0 (7.7)
Fiscal capacity index	0.5 (0.3)	0.5 (0.3)	0.5 (0.3)	0.5 (0.3)
Baseline certification rate (%)	17.6 (4.8)	17.6 (4.7)	17.6 (5.0)	17.7 (3.7)
Community General support centers, mean (SD)	3.7 (7.4)	2.4 (3.4)	3.7 (7.5)	4.1 (8.0)
Early dementia support teams, mean (SD)	1.6 (2.5)	1.4 (1.0)	1.6 (2.5)	1.7 (3.0)
Dementia support promoters, mean (SD)	5.0 (9.1)	3.8 (3.7)	5.0 (9.0)	5.7 (10.7)

Values are presented as *n* (%) for categorical variables and means (SDs) for continuous variables. Implementation categories referred to preventive physical activity programs: no implementation = not conducted, partial implementation = planned but partly conducted, and full implementation = planned and fully conducted. Baseline certification rate was the average for 2018–2019

### COVID-19, coronavirus disease

**Fig. 1:**
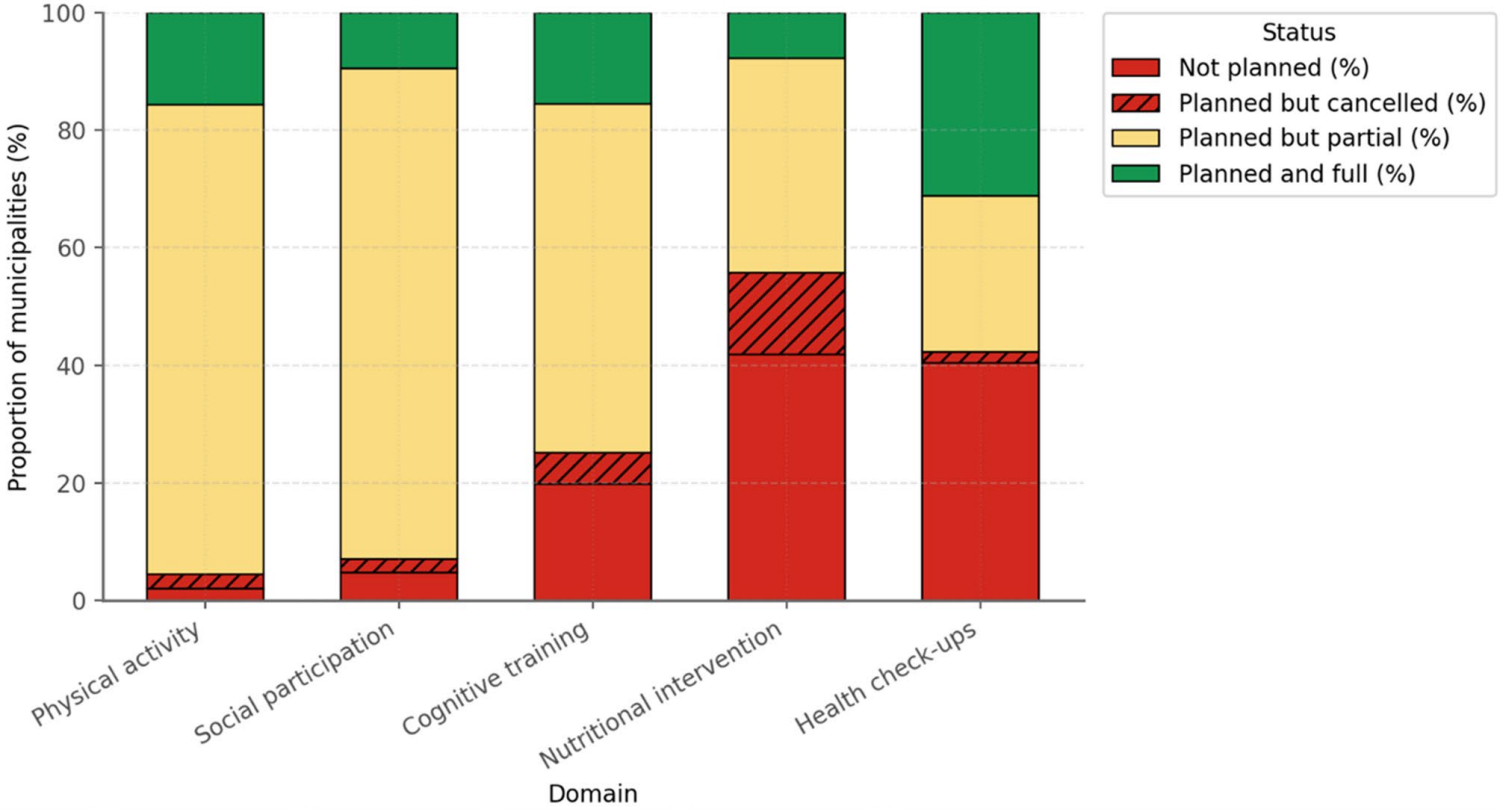
Implementation status of preventive programs (2021, during the COVID-19 pandemic). Stacked bar charts show the proportion of municipalities based on implementation status―not planned, planned but cancelled, planned and partly implemented, and planned and fully implemented―across five domains: physical activity, social participation, cognitive training, nutritional intervention, and health check-ups. Data are obtained from the nationwide survey conducted in 2021, reflecting the situation during the COVID-19 pandemic. COVID-19, Coronavirus disease

During the COVID-19 pandemic period (FY2020–2021), 174 (15.6%) municipalities reported full implementation of physical activity programs, whereas 158 (14.2%) reported full implementation of cognitive training programs. Programs with pandemic-related modifications, such as shorter sessions, restricted attendance, and alternative delivery modes, were classified as “fully implemented” if the planned activities were delivered. Implementation of social participation, nutrition, and health check-up programs was more heterogeneous, with a larger proportion of municipalities reporting partial implementation or outright cancellation (Fig. 1).

**Fig. 2:**
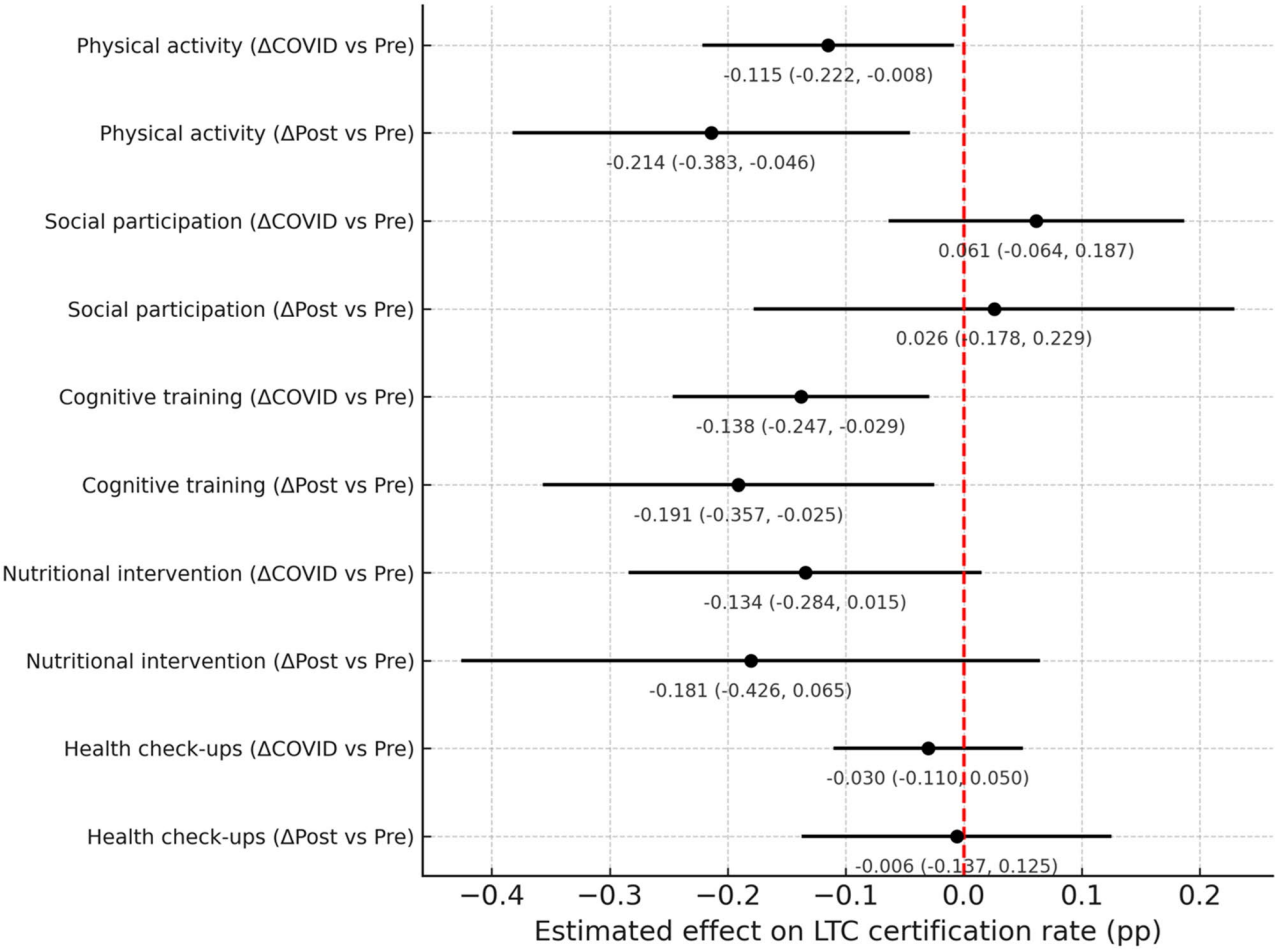
Collapsed DiD estimates for preventive program implementation (full vs. incomplete, 2021 nationwide survey). Estimates were derived from collapsed DiD models. Contrasts are defined as ΔCOVID = (mean of 2020–2021) – (mean of 2018–2019) and ΔPost = (mean of 2022–2023) – (mean of 2018–2019). Models are adjusted for baseline level (2018–2019 mean), aging rate, log(population), and fiscal capacity index. Standard errors are clustered at the municipality level. COVID-19, Coronavirus disease; DiD, Difference-in-differences; LTC, Long-term care

In the primary collapsed DiD analyses, full implementation of physical activity programs was associated with smaller increases in LTC certification rates during the COVID-19 pandemic period compared with incomplete implementation (-0.12 pp, 95%CI: -0.23–-0.01) (Fig. 2). Full implementation of cognitive training programs showed a comparable association (-0.14 pp, 95%CI: -0.25–-0.03). No consistent associations with social participation, nutrition, or health check − ups were observed.

**Fig. 3:**
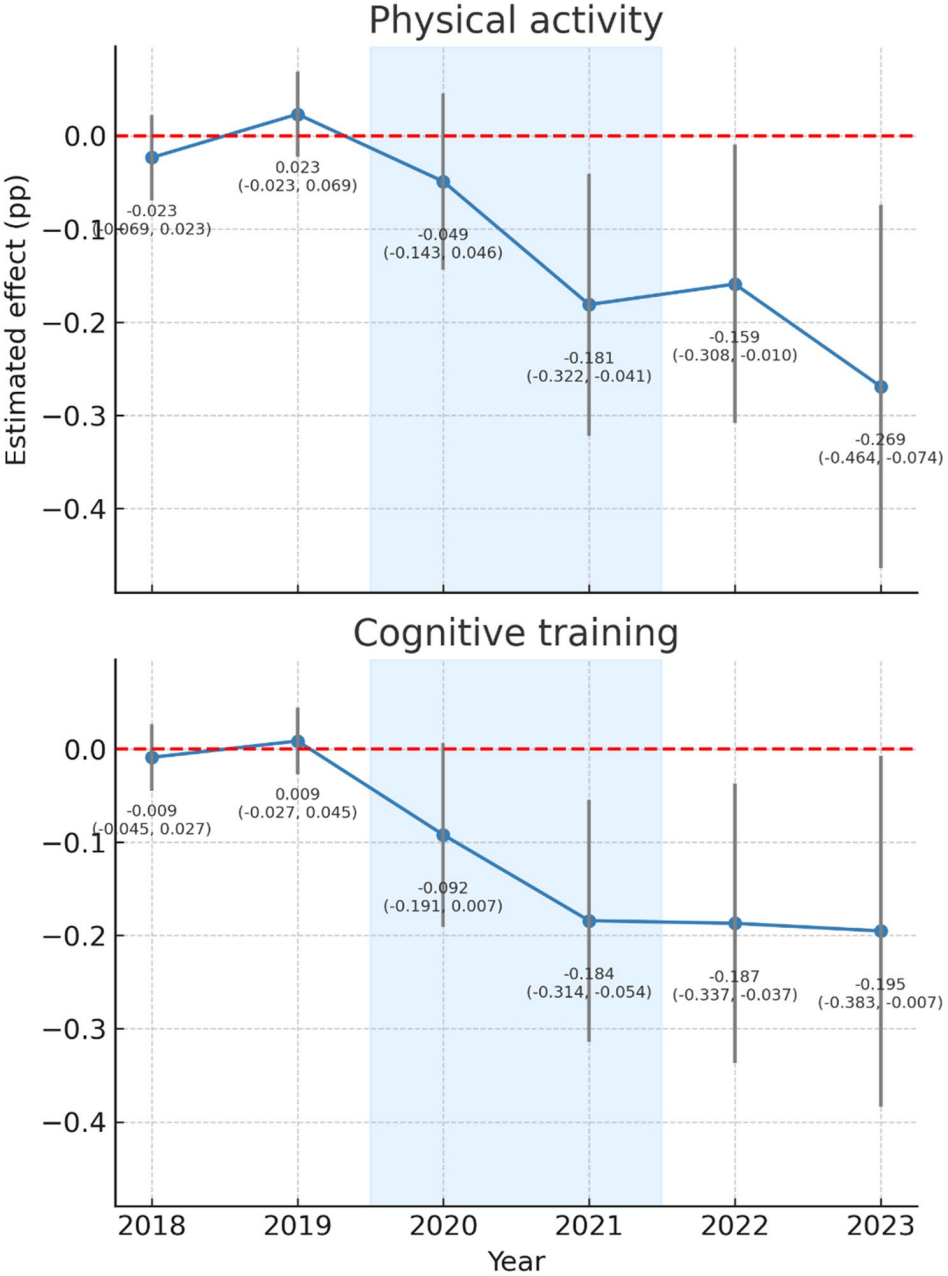
Estimates for physical activity and cognitive training programs (full vs. incomplete implementation, 2021 nationwide survey). Event-study estimates represent year-specific differences in LTCI certification-rate changes between municipalities with full versus incomplete implementation. Outcomes are expressed as yearly deviations from the pre-pandemic baseline, defined as the mean LTCI certification rate in FY2018–2019. Models are adjusted for baseline LTCI certification rate, aging rate, log-transformed population size, and fiscal capacity index. Negative values indicate smaller increases in LTCI certification rates among municipalities with full implementation than among those with incomplete implementation. COVID-19, Coronavirus disease; LTCI, Long-term care insurance

Event−study estimates, expressed as deviations from the pre−pandemic baseline (mean of FY2018–2019), showed that differences associated with physical activity and cognitive training programs were also observed in the post-pandemic period (Fig. 3). Estimated differences for physical activity programs were − 0.18 pp (95%CI: -0.32–-0.04) in FY2021, -0.16 pp (-0.31–-0.01) in FY2022, and − 0.27 pp (-0.46–-0.07) in FY2023. Cognitive training programs showed a similar pattern, with estimates of -0.18 pp (-0.31–-0.05) in FY2021, -0.19 pp (-0.34–-0.04) in FY2022, and − 0.20 pp (-0.38–-0.01) in FY2023. Year-specific estimates for social participation, nutrition, and health check-ups were inconsistent, with confidence intervals crossing the null. Detailed event-study estimates are shown in Table S1.

Sensitivity analyses yielded broadly similar directional patterns, although the estimates were modest and less precise in several models. In models additionally adjusted for municipal service infrastructure and prefecture-level COVID-19 burden, full implementation of physical activity programs remained associated with smaller post-pandemic increases in LTCI certification rates (-0.192 pp, 95%CI: -0.367–-0.017), whereas the estimate for cognitive training was in the same direction but less precise (-0.154 pp, 95%CI: -0.328–0.020) (Table S2). After excluding municipalities that had not planned each program at the beginning of FY2020, estimates for physical activity and cognitive training remained negative, but confidence intervals crossed the null in several contrasts (Table S3). Analyses using alternative LTCI certification outcomes showed smaller and less precise estimates compared with those observed for the primary outcome. Directionally similar estimates were observed for physical activity and cognitive training, particularly in the post-pandemic period, whereas estimates for other domains were less consistent across outcomes and periods (Table S4). A comparison of responding and non-responding municipalities showed that responding municipalities had larger populations, lower aging rates, and higher fiscal capacity indices, whereas baseline LTCI certification rates were similar between groups (Table S5).

## Discussion

In this nationwide ecological analysis of municipal-level preventive service delivery and administrative disability outcomes, full implementation of physical activity and cognitive training programs planned for FY2020 was associated with smaller subsequent increases in LTCI certification rates. These associations were not observed consistently for social participation, nutrition, or health check-ups. Sensitivity analyses generally showed similar directional patterns, although estimates were modest and less precise after additional contextual adjustment, exclusion of municipalities without planned programs, and inclusion of alternative LTCI certification outcomes. These findings should therefore be interpreted as adjusted municipal-level associations rather than causal effects of program implementation.

The associations observed for physical activity are consistent with prior evidence linking physical frailty to subsequent disability among community-dwelling older adults [30], as well as with the rationale for including physical activity components in long-term care prevention programs [26]. Differences were modest during the acute phase of the pandemic and appeared larger in subsequent years, although this temporal pattern should be interpreted cautiously. Cognitive training programs showed a similar pattern, aligning with previous trials reporting sustained cognitive performance following structured training, including multidomain interventions [15]. Although the present ecological design precludes inference at the individual level, these patterns are compatible with the possibility that programs targeting functional and cognitive capacity may be associated with downstream administrative disability outcomes. The sensitivity analyses using alternative outcome definitions suggested that the observed associations were clearer for the broader LTCI certification outcome, which included Support Level 1 or higher, than for more severe certification thresholds. This pattern may indicate that short-term differences in community-based preventive activity are more readily reflected in the earlier or milder care-need certification than in more severe disability categories within the observed follow-up period. However, because this study did not include individual-level information on participation, program dose, or functional change, this interpretation remains tentative.

Associations for the remaining domains were less consistent. In Japan, many social participation programs, including kayoi-no-ba and related community-based activities, are delivered through in-person group formats [31]. Recent evidence also suggests that participation in kayoi-no-ba during the COVID-19 pandemic was associated with favorable frailty outcomes 1 year later [32]. Therefore, the inconsistent association observed for the social participation domain in the present study should not be interpreted as evidence against the potential benefits of individual participation. Instead, our municipal-level exposure may not have reflected participant reach, attendance, dose, or fidelity. National guidance during the COVID-19 pandemic emphasized avoidance of the “three Cs” and identified “five situations” associated with increased infection risk, which likely constrained the delivery and attendance of such activities [18, 19]. Although digital interventions have been proposed as alternatives for reducing loneliness and social isolation among older adults, uptake may have been limited by lower internet use among the oldest age groups and by the in-person orientation of many municipal activities [33, 34]. Nutritional activities target intermediate risk factors, such as dietary diversity and frailty, and may require sustained participation before changes are reflected in LTCI certification outcomes [35]. Health check-ups are primarily designed for risk detection and guidance rather than direct prevention of functional decline; following service interruptions, their resumption may have also have affected detection or referral pathways [36]. In the alternative outcome analyses, some estimates for nutritional intervention were negative, but these patterns were not consistent across periods or severity thresholds. Because the present data did not measure participant reach, fidelity, or actual attendance, domain-specific interpretations should remain tentative.

The observed variation in program implementation also has implications for emergency preparedness in community-based prevention. In the Japanese LTCI system, municipalities are responsible for translating national program guidelines into local delivery, and differences in implementation during the pandemic may have reflected variation in organizational resources, staffing flexibility, leadership, community partnerships, or prior experience with service adaptation [37]. This interpretation is consistent with implementation science frameworks emphasizing the role of organizational context, including available resources, implementation climate, and readiness for change, in sustaining service delivery [38, 39]. From a Reach, Effectiveness, Adoption, Implementation, and Maintenance perspective, the present study relates most directly to the implementation and maintenance dimensions under conditions of external stress [40]. However, these implementation mechanisms were not directly measured. Therefore, the present findings should be viewed as population-level evidence that municipal implementation status during the pandemic was associated with subsequent LTCI certification trends, rather than as evidence identifying specific implementation strategies responsible for those trends.

The finding that full implementation was reported by only 15–16% of municipalities for physical activity and cognitive training highlights the challenge of maintaining community-based programs during systemic disruptions. This result is consistent with international evidence on the vulnerability of locally delivered, group-based health services to pandemic-related constraints [41], and suggests that resilience of preventive service systems cannot be expected even in well-established institutional settings. Whether the observed variation reflected differences in municipal managerial capacity, fiscal resources, community engagement, or a combination of factors could not be determined from the present data but represents an avenue for future implementation research. Recent studies have also suggested that municipal prevention efforts and participation in community gathering places may have heterogeneous associations with older-adult outcomes across local contexts, indicating that adjustment for population size alone may not fully reflect contextual variation [42, 43].

Although rooted in the Japanese LTCI system, these findings have broader relevance for health systems in which community-based preventive services for older adults are organized at the subnational level. Across OECD countries, long-term care systems increasingly emphasize prevention and community-based service delivery to manage rising demand driven by population aging. Germany’s Pflegestärkungsgesetz reforms and Australia’s Commonwealth Home Support Programme, for example, similarly rely on local or regional authorities to implement prevention-oriented services within national regulatory frameworks [10]. The challenge of maintaining such programs during emergencies is not unique to Japan; the WHO Pulse Surveys documented widespread disruption of preventive and promotive services across high-, middle-, and low-income countries, with services for older adults among those most affected.

Analytical approach was used to examine variation in subnational implementation intensity within a shared national policy context, together with a standardized administrative outcome. Countries operating needs-assessment-based LTC eligibility systems (e.g., Germany, the Republic of Korea, and the Netherlands) could apply similar methods to evaluate the downstream consequences of service disruptions, provided that administrative outcome data are available at the relevant geographic level. More broadly, the findings contribute to the emerging evidence base on the indirect health consequences of pandemic-related service disruption [44], extending the focus from clinical services (such as cancer screening or vaccination) to community-based preventive programs whose effects unfold over longer time horizons.

From a policy perspective, these findings suggest the importance of planning for feasible implementation of the community-based preventive services during emergencies. While business continuity plans for healthcare facilities are well established, their extension to community-based preventive services, which are typically lower in priority hierarchies during acute crises, remains underdeveloped in many systems [45, 46]. Municipalities that were able to implement physical activity and cognitive training programs during the pandemic may have benefited from pre-existing organizational capacity for adaptation, such as flexible staffing, community partnerships, and prior investment in alternative delivery modalities.

These findings suggest several considerations for preparedness planning. First, municipalities and subnational authorities may consider prioritizing feasible implementation of physical activity and cognitive training programs during disruptions, given the domain-specific pattern observed here and the supporting evidence base for their efficacy in maintaining functional capacity [26, 30]. Second, investment in delivery adaptations, including smaller group formats, outdoor and well-ventilated settings, and hybrid or digitally supported programming [47], may help support the resilience of community-based prevention systems to future disruptions, although attention to digital literacy and access among the oldest age groups is essential [34, 45]. Third, central governments could support local preparedness by developing operational guidance specific to preventive services under emergency conditions, analogous to the clinical service continuity frameworks issued by World Health Orgaization and national health authorities during the COVID-19 pandemic [48].

This study had some limitations. First, the ecological design precluded individual-level adjustment and causal inference. Municipalities that fully implemented preventive programs may have differed from those with incomplete implementation in unmeasured administrative capacity, community resources, health service use, or other area-level determinants of disability. Second, exposure classifications were self-reported by municipalities and may have been subject to misclassification. Dichotomizing implementation status as “full” versus “incomplete” may have obscured heterogeneity within the latter category, which included partial implementation, cancellation, and programs not planned at the start of FY2020. To address this, we conducted a sensitivity analysis excluding municipalities that had not planned each program. The results were consistent with the primary analysis but less precise and should therefore be interpreted with caution. Third, implementation status was measured at a single survey point and referred to the implementation of programs planned for FY2020. We did not observe annual program availability or implementation intensity throughout FY2018–2023. Therefore, the exposure should be interpreted as FY2020 municipal implementation status rather than longitudinal continuity across the full study period. Fourth, LTCI certification rates may reflect not only changes in underlying functional status but also pandemic-related disruptions in administrative processes, including delays in assessor-administered evaluations, modifications to certification committee operations, and shifts in application behavior among older adults and families. Municipalities able to maintain preventive programs may also have preserved more stable certification procedures. Because our data did not include information on assessment delays, application rates, committee operations, or approval practices, we were unable to distinguish changes in functional need from those in administrative measurement. We also did not account for mortality or competing risks, which may influence municipal LTCI certification rates by changing the population at risk. Future studies should examine LTCI certification, mortality, and functional outcomes jointly. Fifth, the survey response rate was 64.3%, raising the possibility of nonresponse bias. In supplementary analyses, responding municipalities had larger populations, lower aging rates, and higher fiscal capacity indices than non-responding municipalities, although baseline LTCI certification rates were similar. These differences suggest that responding municipalities may have had greater administrative or fiscal capacity, which could limit generalizability and bias estimates if these characteristics were also associated with program implementation and LTCI certification trends. Finally, the study did not capture individual-level participation, program reach, dose, fidelity, or the content and quality of adapted delivery formats. Future studies should link municipal implementation data with individual-level participation and certification data and incorporate detailed measures of implementation processes.

## Conclusions

In conclusion, full implementation of physical activity and cognitive training programs planned for FY2020 was associated with smaller subsequent increases in municipal LTCI certification rates. These associations were supported by sensitivity analyses but were modest and less precise under alternative exposure and outcome definitions. The findings suggest that municipal implementation during the COVID-19 pandemic may be related to population-level care-need trends; however, causal interpretation is limited by the ecological design, single-time assessment of implementation status, potential exposure misclassification, and pandemic-related changes in LTCI certification procedures.

## Supplementary Information

Below is the link to the electronic supplementary material.

Supplementary Material 1

## Data Availability

Annual long-term care (LTC) certification statistics are publicly available from the Ministry of Health, Labor and Welfare Long-Term Care Insurance Status Report. The municipal survey dataset contains municipality-level aggregated information and is not publicly archived. De-identified survey data, questionnaire (Japanese original and English translation), and analytic codes are available from the corresponding author upon reasonable request.

## Abbreviations

LTC: Long-term care
LTCI: Long-term care insurance
COVID-19: Coronavirus disease 2019
FY: Fiscal year
DiD: Difference-in-differences
CI: Confidence interval; pp, percentage point
MHLW: Ministry of Health, Labor and Welfare
