# Supplementary material for: Municipal implementation of community-based preventive programs during the COVID-19 pandemic and long-term care certification trends in Japan: a nationwide ecological analysis
Source: BMC Health Serv Res. 2026 Jun 6;26:1096. doi: 10.1186/s12913-026-14909-0 (PMC13466528; doi:10.1186/s12913-026-14909-0)
Supplement: Supplementary file 1 [file 12913_2026_14909_MOESM1_ESM.docx]

Table S1: Event-study estimates based on year and domain (full vs. incomplete implementation)

| **Domain** | **Year** | **Estimate (pp)** | **95%CI** |
| --- | --- | --- | --- |
| Physical activity | 2018 | −0.023 | (−0.069‒0.023) |
|  | 2019 | 0.023 | (−0.023‒0.069) |
|  | 2020 | −0.049 | (−0.143‒0.046) |
|  | 2021 | −0.181 | (−0.322‒ −0.041) |
|  | 2022 | −0.159 | (−0.308‒ −0.01) |
|  | 2023 | −0.269 | (−0.464‒ −0.074) |
| Social participation | 2018 | −0.032 | (−0.082‒0.018) |
|  | 2019 | 0.032 | (−0.018‒0.082) |
|  | 2020 | 0.003 | (−0.104‒0.11) |
|  | 2021 | 0.120 | (−0.039‒0.279) |
|  | 2022 | 0.052 | (−0.132‒0.237) |
|  | 2023 | −0.001 | (−0.229‒0.227) |
| Cognitive training | 2018 | −0.009 | (−0.045‒0.027) |
|  | 2019 | 0.009 | (−0.027‒0.045) |
|  | 2020 | −0.092 | (−0.191‒0.007) |
|  | 2021 | −0.184 | (−0.314‒ −0.054) |
|  | 2022 | −0.187 | (−0.337‒ −0.037) |
|  | 2023 | −0.195 | (−0.383‒ −0.007) |
| Nutritional intervention | 2018 | −0.015 | (−0.066‒0.036) |
|  | 2019 | 0.015 | (−0.036‒0.066) |
|  | 2020 | −0.128 | (−0.265‒0.009) |
|  | 2021 | −0.141 | (−0.317‒0.036) |
|  | 2022 | −0.138 | (−0.351‒0.075) |
|  | 2023 | −0.224 | (−0.51‒0.063) |
| Health check-ups | 2018 | 0.005 | (−0.027‒0.037) |
|  | 2019 | −0.005 | (−0.037‒0.027) |
|  | 2020 | −0.030 | (−0.1‒0.04) |
|  | 2021 | −0.031 | (−0.132‒0.071) |
|  | 2022 | 0.001 | (−0.119‒0.12) |
|  | 2023 | −0.012 | (−0.162‒0.137) |

**Footnote:** Event-study models regressed the annual deviation from the pre-coronavirus disease baseline (yearly certification rate minus mean FY2018-2019) on a full-implementation indicator, adjusting for aging rate, log-transformed population size, and fiscal capacity index. Negative estimates indicate smaller increases in certification rates among municipalities with full implementation than among those with incomplete implementation. Robust standard errors are used.

Table S2. Sensitivity analysis additionally adjusted for municipal service infrastructure and prefecture-level COVID-19 burden

| **Domain** | **Contrast** | **N** | **Full implementation, n** | **Estimate, pp** | **95%CI** | **p-value** |
| --- | --- | --- | --- | --- | --- | --- |
| Physical activity | COVID vs. Pre | 1,111 | 175 | -0.076 | -0.181 to 0.029 | 0.157 |
| Physical activity | Post vs. Pre | 1,111 | 175 | -0.192 | -0.367 to -0.017 | 0.031 |
| Social participation | COVID vs. Pre | 1,111 | 106 | 0.047 | -0.110 to 0.204 | 0.559 |
| Social participation | Post vs. Pre | 1,111 | 106 | 0.050 | -0.217 to 0.317 | 0.713 |
| Cognitive training | COVID vs. Pre | 1,111 | 172 | -0.076 | -0.184 to 0.032 | 0.167 |
| Cognitive training | Post vs. Pre | 1,111 | 172 | -0.154 | -0.328 to 0.020 | 0.082 |
| Nutritional intervention | COVID vs. Pre | 1,111 | 88 | -0.071 | -0.216 to 0.075 | 0.341 |
| Nutritional intervention | Post vs. Pre | 1,111 | 88 | -0.161 | -0.424 to 0.102 | 0.229 |
| Health check-ups | COVID vs. Pre | 1,111 | 346 | -0.033 | -0.115 to 0.049 | 0.428 |
| Health check-ups | Post vs. Pre | 1,111 | 346 | -0.004 | -0.144 to 0.136 | 0.957 |

**Footnote:** Estimates represent percentage-point differences in changes in LTCI certification rates between municipalities with full versus incomplete implementation, using the main exposure definition. COVID vs. Pre is defined as the mean FY2020-2021 certification rate minus the mean FY2018-2019 certification rate. Post vs. Pre is defined as the mean FY2022-2023 certification rate minus the mean FY2018-2019 certification rate. Models were adjusted for baseline LTCI certification rate, aging rate, log-transformed population size, fiscal capacity index, community general support centers per 100,000 population, and prefecture-level COVID-19 burden. Robust standard errors were used.

COVID-19, coronavirus disease

Table S3. Sensitivity analysis excluding municipalities that had not planned each preventive program at the beginning of FY2020

| **Domain** | **Contrast** | **N** | **Full implementation, n** | **Estimate, pp** | **95% CI** | **p-value** |
| --- | --- | --- | --- | --- | --- | --- |
| Physical activity | COVID vs. Pre | 1,083 | 172 | -0.088 | -0.200 to 0.023 | 0.120 |
| Physical activity | Post vs. Pre | 1,083 | 172 | -0.171 | -0.353 to 0.011 | 0.066 |
| Social participation | COVID vs. Pre | 1,061 | 105 | 0.032 | -0.132 to 0.196 | 0.702 |
| Social participation | Post vs. Pre | 1,061 | 105 | 0.067 | -0.199 to 0.333 | 0.622 |
| Cognitive training | COVID vs. Pre | 895 | 172 | -0.110 | -0.222 to 0.003 | 0.055 |
| Cognitive training | Post vs. Pre | 895 | 172 | -0.152 | -0.328 to 0.025 | 0.092 |
| Nutritional intervention | COVID vs. Pre | 656 | 84 | -0.113 | -0.279 to 0.053 | 0.181 |
| Nutritional intervention | Post vs. Pre | 656 | 84 | -0.157 | -0.432 to 0.119 | 0.264 |
| Health check-ups | COVID vs. Pre | 673 | 342 | -0.023 | -0.115 to 0.069 | 0.626 |
| Health check-ups | Post vs. Pre | 673 | 342 | 0.008 | -0.143 to 0.158 | 0.922 |

**Footnote:** This analysis is restricted to municipalities that had planned each preventive program at the beginning of FY2020. Municipalities that reported “not planned” for the relevant domain are excluded. The comparison is therefore between municipalities with full implementation and those with partial implementation, cancellation, or other non-full implementation among municipalities with planned programs. Models are adjusted for baseline LTCI certification rate, aging rate, log-transformed population size, and fiscal capacity index. Robust standard errors are used. When a response included “not planned” together with another category, the record is excluded from this sensitivity analysis for the relevant domain.

COVID-19, coronavirus disease; LTCI, long-term care insurance

Table S4. Sensitivity analysis using alternative LTCI certification outcome definitions

| **Outcome** | **Domain** | **Contrast** | **N** | **Full implementation, n** | **Estimate, pp** | **95% CI** | **p-value** |
| --- | --- | --- | --- | --- | --- | --- | --- |
| Care Level 1 or higher | Physical activity | COVID vs. Pre | 1,113 | 175 | -0.073 | -0.171 to 0.025 | 0.145 |
| Care Level 1 or higher | Physical activity | Post vs. Pre | 1,113 | 175 | -0.177 | -0.321 to -0.032 | 0.016 |
| Care Level 1 or higher | Social participation | COVID vs. Pre | 1,113 | 106 | 0.018 | -0.097 to 0.133 | 0.759 |
| Care Level 1 or higher | Social participation | Post vs. Pre | 1,113 | 106 | -0.006 | -0.180 to 0.167 | 0.942 |
| Care Level 1 or higher | Cognitive training | COVID vs. Pre | 1,113 | 173 | -0.061 | -0.146 to 0.024 | 0.157 |
| Care Level 1 or higher | Cognitive training | Post vs. Pre | 1,113 | 173 | -0.117 | -0.248 to 0.015 | 0.082 |
| Care Level 1 or higher | Nutritional intervention | COVID vs. Pre | 1,113 | 88 | -0.124 | -0.276 to 0.027 | 0.107 |
| Care Level 1 or higher | Nutritional intervention | Post vs. Pre | 1,113 | 88 | -0.213 | -0.417 to -0.010 | 0.040 |
| Care Level 1 or higher | Health check-ups | COVID vs. Pre | 1,113 | 348 | -0.008 | -0.080 to 0.064 | 0.827 |
| Care Level 1 or higher | Health check-ups | Post vs. Pre | 1,113 | 348 | 0.000 | -0.112 to 0.113 | 0.995 |
| Care Level 2 or higher | Physical activity | COVID vs Pre | 1,113 | 175 | -0.042 | -0.125 to 0.041 | 0.324 |
| Care Level 2 or higher | Physical activity | Post vs Pre | 1,113 | 175 | -0.108 | -0.229 to 0.013 | 0.079 |
| Care Level 2 or higher | Social participation | COVID vs. Pre | 1,113 | 106 | 0.033 | -0.067 to 0.134 | 0.516 |
| Care Level 2 or higher | Social participation | Post vs. Pre | 1,113 | 106 | 0.018 | -0.121 to 0.158 | 0.799 |
| Care Level 2 or higher | Cognitive training | COVID vs. Pre | 1,113 | 173 | -0.043 | -0.119 to 0.033 | 0.266 |
| Care Level 2 or higher | Cognitive training | Post vs. Pre | 1,113 | 173 | -0.091 | -0.200 to 0.018 | 0.102 |
| Care Level 2 or higher | Nutritional intervention | COVID vs. Pre | 1,113 | 88 | -0.058 | -0.172 to 0.055 | 0.312 |
| Care Level 2 or higher | Nutritional intervention | Post vs. Pre | 1,113 | 88 | -0.119 | -0.262 to 0.024 | 0.104 |
| Care Level 2 or higher | Health check-ups | COVID vs. Pre | 1,113 | 348 | -0.010 | -0.075 to 0.056 | 0.767 |
| Care Level 2 or higher | Health check-ups | Post vs. Pre | 1,113 | 348 | -0.018 | -0.114 to 0.078 | 0.713 |

**Footnote:** The main analysis used the total LTCI certification rate, defined as certification at Support Level 1 or higher. This sensitivity analysis usea two alternative outcome definitions: certification at Care Level 1 or higher and certification at Care Level 2 or higher. Models are adjusted for the baseline value of the corresponding LTCI certification outcome, aging rate, log-transformed population size, and fiscal capacity index. Estimates are shown as percentage-point differences. Robust standard errors are used.

LTCI, long-term care insurance

Table S5. Comparison of responding and non-responding municipalities

| **Characteristic** | **Responding municipalities** | **Non-responding municipalities** | **Standardized mean difference** | **p-value** |
| --- | --- | --- | --- | --- |
| Municipalities, n | 1,110 | 631 | — | — |
| Population, mean (SD) | 88,680.4 (222,691.3) | 43,915.8 (109,101.5) | 0.255 | <0.001 |
| Aging rate (%), mean (SD) | 34.03 (7.87) | 36.01 (7.89) | -0.252 | <0.001 |
| Fiscal capacity index | 0.512 (0.270) | 0.436 (0.263) | 0.287 | <0.001 |
| Baseline LTCI certification rate, 2018-2019 mean (%) | 17.888 (2.713) | 17.931 (2.968) | -0.015 | 0.779 |

**Footnote:** Responding municipalities are identified from the 2021 municipal survey dataset. Non-responding municipalities are defined as municipalities listed in the national municipal fiscal dataset but not identified as survey respondents. Population and aging rate are obtained from the municipal population statistics dataset. Baseline LTCI certification rate is calculated as the mean of FY2018 and FY2019 certification rates. The fiscal capacity index is obtained from the national municipal fiscal indicators file. p-values are calculated using Welch’s t-tests. This comparison is conducted to assess the possibility of nonresponse bias.

LTCI, long-term care insurance
